# Immunosuppressant Responsive Neuropsychiatric Systemic Lupus Erythematosus Manifestations Initially Diagnosed As Schizophrenia and Bipolar Disorder

**DOI:** 10.7759/cureus.29287

**Published:** 2022-09-18

**Authors:** Benjamin D Goodman, Tala Al-Saghir, Harsha Alles, Rami M Youssef, Hesham Shaban

**Affiliations:** 1 Internal Medicine, Henry Ford Health, Detroit, USA; 2 Division of Nephrology and Hypertension, Henry Ford Health, Detroit, USA; 3 Division of Palliative Care, Henry Ford Health, Detroit, USA

**Keywords:** systemic lupus erythematosus, sle, bipolar, schizophrenia, psychosis, cyclophosphamide, neuropsychiatric sle

## Abstract

Systemic lupus erythematosus (SLE) is a well-documented multi-system autoimmune disease with increased frequency noted in younger females and among minority populations. Disease-defining signs and symptoms can vary widely and involve multiple organ systems including the nervous system. Involvement of the nervous system, known as neuropsychiatric SLE (NPSLE) can present as manifestations consistent with central nervous system or peripheral nervous system pathologies, with the former including presentations of psychiatric illnesses. This case report reviews a 21-year-old Black female’s presentation that was most notable for psychosis with other findings on examination and laboratory investigation resulting in a diagnosis of NPSLE. Our patient had a positive initial response to high-dose steroids with improvement of her psychosis and was planned for further treatment with the well-known chemotherapy and immunomodulatory agent, cyclophosphamide.

## Introduction

Systemic lupus erythematosus (SLE) is a multisystem autoimmune disorder that can affect any organ and is the most common type of lupus [1]. Due to its ability to affect any organ, SLE can include a multitude of symptoms; some of the most common symptoms include fatigue, fever, skin rashes, and joint pain. In severe and prolonged cases, SLE can lead to organ failure. SLE is more commonly seen in females than males by a ratio of 8-15:1. Individuals typically present with symptoms of SLE between the ages of 15-44 [1,2]. A greater prevalence of SLE is noted among women of minority ethnicities and races such as American Native, Asian, Black, Hispanic, and Latino [1]. In the United States, SLE has an incidence of 5.1 per 100,000 and an estimated prevalence of 97.4 per 100,000 [1,3]. 

When SLE presents with symptoms of the nervous system, it is termed Neuropsychiatric SLE (NPSLE). NPSLE is often an early manifestation in the course of SLE and can precede other symptoms by several years. To date, research suggests that the incidence of NPSLE in SLE patients is 30-40% [4]. In the time period from six months prior to diagnosis of SLE and up to 15 months after, approximately 39% of patients will have at least one NPSLE symptom. This can include but is not limited to cognitive dysfunction (6.6-80%), mood disorders (7.4-65%), anxiety disorders (6.4-40%), psychosis (0.6-11%), acute confusional state (0.9-7%), neuropathies (3.4-12.3%), headaches (12.2-28.3%), and seizures (7.0-20%) [5,6]. 

NPSLE is a diagnosis of exclusion but, at the same time and in the appropriate context, should be considered due to its ability to present with varying symptoms. Other potential etiologies of a patient’s neurologic or psychiatric symptoms may include autoimmune causes, hemorrhage, infectious causes, inflammation, malignancies, myelopathies, neuropathies, seizures, thromboembolic causes, thyroid disease, or vitamin deficiencies [2]. NPSLE diagnosis also necessitates either a current diagnosis of SLE or a future diagnosis of SLE to attribute past events to SLE. Frequently utilized criteria for SLE diagnosis are provided by the American College of Rheumatology and European League Against Rheumatism and include a positive Antinuclear antibodies (ANA) titer of ≥ 1:80 in addition to one clinical criterion and ≥ 10 points based on diagnostic signs, symptoms, laboratory results, and biopsy data [7].

Deciding a specific NPSLE treatment is often dependent on the presenting symptoms. For example, more inflammatory symptoms usually require acute immunosuppression and close outpatient follow-up for continuing long-term immunosuppression. If the symptoms are more thrombotic in nature, modification of thromboembolic or cardiovascular risk factors would be a more beneficial approach. Often, a multidisciplinary approach may be needed for treatment when there is involvement of the nervous system or psychiatric symptoms are present [2].

## Case presentation

A 21-year-old Black female with a past medical history of recently diagnosed schizophrenia, bipolar disorder, and SLE presented to our quaternary care center due to a petition by her mother after increased agitation. Over the nine weeks prior to admission, she had several outside hospital encounters including an emergency department visit for a rash on her hands, feet, and nose, another emergency department visit for agitation, a hospitalization for psychosis where infectious etiologies were ruled out via a lumbar puncture, and finally in-patient psychiatric hospitalization. One week prior to arrival at our facility, her diagnosis of SLE had been made, corresponding diagnostic laboratory tests had been ordered, and treatment with hydroxychloroquine and prednisone had been initiated. On admission to our facility, she endorsed blurred vision, but otherwise was noted to be afebrile, hemodynamically stable apart from tachycardia, and oriented to person, place, time, and event. Further examination was not significant for focal neurological deficits or meningeal signs, but she was found to have a malar-appearing hyperpigmented rash sparing the nasolabial folds, rhythmic tremors in the upper extremities, hyperreflexia in the lower extremities, and a flat affect.

In anticipation of the patient’s SLE diagnosis as a result of her positive ANA test and cutaneous finding, and to better characterize her disease, a thorough SLE workup was conducted. All laboratory tests and results can be found in Table 1. 

**Table 1:** Systemic lupus erythematosus (SLE) laboratory test results

Component	Patient's Value	Reference Range & Units
Antinuclear Antibodies	1:2560	<1:80
Anti-β2 Glycoprotein Antibodies, IgA	<9 SAU	<20 SAU
Anti-β2 Glycoprotein Antibodies, IgM	<9 SMU	<20 SMU
Anti-β2 Glycoprotein Antibodies, IgG	<9 SGU	<20 SGU
Anti-Cardiolipin Antibodies, IgA	<9 APL	<12 APL
Anti-Cardiolipin Antibodies, IgM	<9 MPL	<15 MPL
Anti-Cardiolipin Antibodies, IgG	18.3 GPL	<12.5 GPL
Anti-Double Stranded DNA Antibodies, IgG	1:80	<1:10
Anti-Sjögren's Syndrome-A/Ro Antibodies, IgG	>8.0 EU	<1.0 EU
Anti-Sjögren's Syndrome-B/La Antibodies, IgG	>8.0 EU	<1.0 EU
Anti-Smith Antibodies, IgG	>8.0 EU	<1.0 EU
Anti-Ribonucleoprotein Antibodies, IgG	>8.0 EU	<1.0 EU
C-Reactive Protein	20 mg/dL	<0.5 mg/dL
Complement, C3	77 mg/dL	90-230 mg/dL
Complement, C4	11 mg/dL	10-51 mg/dL
Diluted Russel Viper Venom Time	43 sec	27-45 sec
Erythrocyte Sedimentation Rate	70 mm/hr	<20 mm/hr
International Normalized Ratio	0.94	0.8-1.1
Lupus Anticoagulant	41.7 sec	30.3-43.2 sec
Partial Thromboplastin Time	35 sec	22-36 sec
Prothrombin Time	13.1 sec	11.5-14.5 sec
Urine Creatinine	177 mg/dL	No reference range
Urine Protein	182 mg/dL	No reference range

The differential diagnosis of psychosis in an adult is extensive and a thorough workup was necessary to arrive at NPSLE, a diagnosis of exclusion. An initial complete blood count showed microcytic anemia and further iron studies were consistent with iron deficiency anemia. A peripheral blood smear also noted moderate microcytic anemia, and further identified slight polychromasia without overt schistocytosis, dysplasia, or presence of blasts. A basic metabolic panel showed normal electrolyte levels. Ammonia and blood urea nitrogen, which could lead to encephalopathy when elevated, were normal. Thyroid hormone abnormalities as an etiology were ruled out by normal thyroid stimulating hormone and thyroxine levels. Consideration was given to drugs of abuse, but a urine drug screen was negative. An electroencephalogram was conducted while asleep and awake and showed no epileptiform activity to point to seizures as a cause. Knowing that cerebral vasculitis may also cause an altered mental status, magnetic resonance angiography and magnetic resonance 3D reconstruction did not show any evidence of vascular etiologies. 

The results of a lumbar puncture for cerebrospinal fluid (CSF) analysis showed lymphocytic pleocytosis which can typically be seen in infectious, inflammatory, or autoimmune processes. The CSF glucose level was within normal limits. Further CSF evaluation noted no oligoclonal bands. An additional infectious work-up resulted in a negative culture and negative viral, bacterial, and fungal studies. Neurosarcoidosis was able to be ruled out with negative testing for CSF angiotensin converting enzyme in addition to an unremarkable chest X-ray. MRI with and without contrast helped to rule out further neuro-infectious and inflammatory processes. 

Other infectious etiologies including sexually transmitted diseases were ruled out via blood and serum testing. A mildly decreased serum ceruloplasmin with normal liver function and negative MRI was inconsistent with neuropsychiatric symptoms of Wilson’s disease. Anti-N-methyl-D-aspartate (anti-NMDA) antibodies IgG were also negative, ruling out anti-NMDA receptor encephalitis. Common heavy metals, including lead, mercury, cadmium, and arsenic, were not found to be present during testing. 

A diagnosis of NPSLE was considered, and while a thorough workup was being conducted to rule out infectious and reversible causes prior to initiating treatment appropriate for NPSLE, the patient was agitated, psychotic, and had multiple episodes of stated suicidal ideations in addition to one attempt to harm herself. Despite the utilization of a sitter, her degree of psychosis and agitation required placing her in four-point locking restraints in addition to a rotating schedule of quetiapine, haloperidol, lorazepam, and midazolam. After an extensive workup did not show other reversible causes of the patient’s symptoms, a multidisciplinary decision between the patient’s mother, Internal Medicine, Rheumatology, Neurology, Psychiatry, and Infectious Disease was made to start the patient on one gram methylprednisolone a day for three days followed by 50 milligrams of prednisone each subsequent day. This was in addition to the continuation of her hydroxychloroquine. The high-dose steroids led to a resolution of the patient’s acute psychosis and agitation and her disposition continued to improve during the duration of her hospital course. Prior to discharge, Gynecology was consulted to assist with mitigating the infertility risks of treatment with cyclophosphamide, the first-line long-term therapy of NPSLE. In this regard, Leuprolide was recommended by Gynecology to aid in protecting future fertility and was administered 24 hours prior to her cyclophosphamide dose. After receiving a dose of cyclophosphamide in the hospital, the patient was discharged. Further plans for treatment included a reduction of her prednisone dose to 40mg one week after discharge and continuation of this dosage until her follow-up appointment with Rheumatology. Further management of her treatment with cyclophosphamide and leuprolide were to be managed by Rheumatology and Gynecology, respectively. 

## Discussion

A diagnosis of SLE is multifactorial and hinges on the assessment of immune markers, constitutional signs, mucocutaneous and organ manifestations, hematologic cytopenias, and psychiatric involvement. ANA positive serology, the desired prerequisite for SLE diagnosis, carries a 96-99% sensitivity. The less sensitive but highly specific markers anti-Smith (anti-SM) antibodies and anti-double stranded DNA (anti-dsDNA) antibodies are also important diagnostic markers. Due to SLE’s pathogenesis as an immune complex disease, C3, and C4 serum levels can additionally be utilized as markers in SLE; because C3 and/or C4 can be low in a multitude of autoimmune complex diseases, they are not specific for SLE but can aid in monitoring disease progression [8]. 

Mucocutaneous manifestations such as malar rash, maculopapular rash, oral ulcers, alopecia, photosensitivity, and rosacea are often heavily relied on as components of SLE diagnosis due to their distinct clinical presentation. Less specific signs include constitutional symptoms such as fever, arthralgia, myalgia, fatigue, lymphadenopathy, and hematological cytopenias such as autoimmune hemolytic anemia. Organ manifestations such as lupus nephritis must be explored due to their specificity to SLE but also because they are strong indicators of disease progression and severity [8]. As seen in our patient and discussed below, neuropsychiatric manifestations, such as psychosis, can often be present early in SLE’s course. 

Our patient’s labs yielded positive ANA, anti-SM antibodies, and anti-dsDNA antibodies, helping to confirm a diagnosis and opening the door for further definitive SLE evaluation and management. C4 was within normal range, but C3 levels were low indicating complex deposition. Labs for anti-Sjögren's Syndrome A/Ro antibodies were also positive, whereas testing for anti-Sjögren's Syndrome B/La antibodies, anti-β2 glycoproteins antibodies, anti-cardiolipin antibodies, anti-ribosomal P antibodies, and lupus anticoagulant came back negative. Although these autoantibodies can be potential markers for SLE, their negative status does not detract from the diagnosis due to their lack of specificity for SLE and low weight compared to the high weight of ANA, anti-SM antibodies, anti-dsDNA antibodies, and low C3 [8]. While our patient did display the classic malar rash, one of the first clear physical manifestations that prompted further exploration into SLE diagnosis, she presented with no constitutional symptoms. She was noted to have an elevated erythrocyte sedimentation rate, a non-specific indicator of inflammation. Urine studies indicated some potential degree of renal involvement via elevated creatinine and protein levels, but neither a renal biopsy nor a 24-hour urine collection was performed. This culmination of laboratory results and manifestations gave her an American College of Rheumatology and European League Against Rheumatism SLE criteria score of 18 and led to the initiation of hydroxychloroquine and prednisone [7].

The pathogenesis of NPSLE is still not fully understood, but current research implicates multiple autoantibodies that are being further explored in order to produce targeted clinical therapies. Preclinical studies show that anti-NR2 (anti-NMDA receptor 2) autoantibodies, antibodies to the NR2 portion of the NMDA receptors, and anti-ribosomal P antibodies are neurotoxic in NPSLE patients once they have crossed the blood-brain barrier (BBB). Anti-NR2 was specifically found to aid in BBB disruption and entry and linked to spatial memory impairment [9]. Serum anti-ribosomal P antibodies were positively correlated with worse outcomes in patients with diffuse NPSLE and are associated with symptoms of psychosis and depression [9,10]. 

In 50-60% of patients, neuropsychiatric manifestations develop within one year of SLE onset [4]. In the case of our patient, her symptoms were within two months of diagnosis. Neuropsychiatric symptoms can include but are not limited to psychosis, seizures, delirium, mononeuritis multiplex, myelitis, peripheral neuropathy, cranial nerve disorders, anxiety, headaches, and acute confusional state [11]. Several screening tools are used to evaluate cognitive dysfunction, anxiety, and depression in SLE patients with psychiatric symptoms, starting with MRI. MRI is considered the gold standard for neuropsychiatric assessment, although only about 50% of NPSLE patients have detectable abnormalities, as exemplified in our patient with a normal MRI [9]. While neuropsychiatric symptoms are not uncommon in SLE, psychosis is one of the less frequently seen manifestations, reported in only 0.6-11% of NSPLE patients [6]. 

Anti-ribosomal P antibodies are also commonly screened in suspected NPSLE patients due to their multifactorial contribution to the blood-brain barrier and increased pro-inflammatory cytokine production [11]. Our patient’s negative anti-ribosomal P antibodies result does not rule out NPSLE as it is not required for the diagnosis. Instead, it may act as a marker for disease severity and long-term disease outlook. We can therefore infer that a negative result could be indicative of a mild long-term disease course for our patient.

A universally agreed upon regimen has not yet been dictated for NPSLE treatment due to the diversity of symptom presentation and limited diagnostic testing modalities, resulting in differing management techniques. Therefore, a commonly used approach to selecting treatment is to classify the disease course as ischemic or inflammatory. While antipsychotics and antidepressants are mainstays for symptomatic psychiatric treatment, aspirin, glucocorticoids, and hydroxychloroquine are the widely used SLE treatments [10]. Of these three, glucocorticoids are the primary treatment for NPSLE but present their own problems due to the potential for resultant CNS side effects [9]. Hydroxychloroquine, an antimalarial, is used to protect against thromboembolic events due to its anti-platelet and lipid-lowering abilities. Anticoagulants are similarly used in NPSLE to reduce the risk of thrombosis, particularly in patients with antiphospholipid antibodies. Cyclophosphamide, a cytotoxic immunosuppressant, has historically been used to treat severe CNS involvement in patients with NPSLE. While effective, the drug causes multiple side effects including hemorrhagic cystitis, increased risk for malignancy and opportunistic infection, and ovarian or testicular atrophy [10].

The regimen proposed by Magro-Checa et al. was utilized in the treatment of our patient. Our patient’s disease was classified as inflammatory NPSLE due to her elevated ESR, psychosis, and lack of findings on neuro-imaging. Accordingly, she was started on high-dose glucocorticoids and then transitioned to monthly intravenous cyclophosphamide with a planned duration of 6 months. At the end of this 6 month period, she will be reevaluated for clinical response. If it is deemed that she has had complete resolution of symptoms, azathioprine, mycophenolate, or cyclosporine and oral prednisone would be initiated for maintenance therapy. If instead she has a partial response at that time, then cyclophosphamide would be administered every three months for 18 months, for a total of six additional doses. If the patient has had no response after the initial six months of cyclophosphamide or a worsening of her symptoms, a second-line treatment, rituximab, would be started [2]. Although our patient was placed on cyclophosphamide, the potential for infertility associated with its use was a serious concern. For this reason, the Gynecology team recommended that the patient be started on leuprolide, a gonadotropin-releasing hormone agonist, for fertility preservation. As noted in a meta-review conducted by Clowse et al. the use of gonadotropin-releasing hormone agonists improved the preservation of ovarian function in women with autoimmune diseases or malignancies receiving cyclophosphamide [12]. 

As demonstrated in the literature cited above, the diagnosis and treatment of NPSLE are still under exploration by the medical community due to its mixed presentation and incompletely understood pathogenesis. For this reason, we cannot emphasize enough the importance of a thorough evaluation of a patient with psychiatric symptoms for organic causes prior to the diagnosis of a psychiatric illness. 

## Conclusions

As a result of the patient’s symptoms and established diagnosis of SLE, a diagnosis of NPSLE appropriately explained her psychiatric symptoms after a thorough evaluation for other potential etiologies. The diagnosis of NPSLE is well supported despite negative anti-ribosomal P antibodies and without the results of additional antibodies that in literature have suggested NPSLE. Additionally, the patient’s appropriate response to glucocorticoids and cyclophosphamide and lack of complete response to antipsychotics continue to strengthen the diagnosis. This case further highlights the importance of a thorough evaluation of organic causes of psychiatric symptoms prior to a psychiatric diagnosis. This is even more important in the age of electronic charts and thus the permanence of what is written, as her incorrect diagnosis of bipolar and schizophrenia will always be present regardless of her more appropriate diagnosis of NPSLE.
